# Cd-free Cu-doped ZnInS/ZnS Core/Shell Nanocrystals: Controlled Synthesis And Photophysical Properties

**DOI:** 10.1186/s11671-018-2599-x

**Published:** 2018-06-18

**Authors:** Manpreet Kaur, Ashma Sharma, Murat Olutas, Onur Erdem, Akshay Kumar, Manoj Sharma, Hilmi Volkan Demir

**Affiliations:** 1 Department of Nanotechnology, Sri Guru Granth Sahib World University, Punjab, 140406 India; 2 Department of Electrical and Electronics Engineering, Department of Physics, and UNAM–Institute of Materials Science and Nanotechnology, Bilkent University, 06800 Ankara, Turkey; 3 LUMINOUS! Center of Excellence for Semiconductor Lighting and Displays, School of Electrical and Electronics Engineering, School of Physical and Mathematical Sciences, School of Materials Science and Engineering, Nanyang Technological University, Nanyang Avenue, Singapore, 639798 Singapore; 4 Department of Physics, Abant Izzet Baysal University, 14030 Bolu, Turkey

**Keywords:** Cadmium free, Colloidal quantum dots, Quantum yield, Cu doping, White-light emission, Color properties

## Abstract

Here, we report efficient composition-tunable Cu-doped ZnInS/ZnS (core and core/shell) colloidal nanocrystals (CNCs) synthesized by using a colloidal non-injection method. The initial precursors for the synthesis were used in oleate form rather than in powder form, resulting in a nearly defect-free photoluminescence (PL) emission. The change in Zn/In ratio tunes the percentage incorporation of Cu in CNCs. These highly monodisperse Cu-doped ZnInS CNCs having variable Zn/In ratios possess peak emission wavelength tunable from 550 to 650 nm in the visible spectrum. The quantum yield (QY) of these synthesized Cd-free CNCs increases from 6.0 to 65.0% after coating with a ZnS shell. The CNCs possessing emission from a mixed contribution of deep trap and dopant states to only dominant dopant-related Stokes-shifted emission are realized by a careful control of stoichiometric ratio of different reactant precursors during synthesis. The origin of this shift in emission was understood by using steady state and time-resolved fluorescence (TRF) spectroscopy studies. As a proof-of-concept demonstration, these blue excitable Cu-doped ZnInS/ZnS CNCs have been integrated with commercial blue LEDs to generate white-light emission (WLE). The suitable combination of these highly efficient doped CNCs results led to a Commission Internationale de l’Enclairage (CIE) color coordinates of (0.33, 0.31) at a color coordinate temperature (CCT) of 3694 K, with a luminous efficacy of optical radiation (LER) of 170 lm/W_opt_ and a color rendering index (CRI) of 88.

**Electronic supplementary material:**

The online version of this article (10.1186/s11671-018-2599-x) contains supplementary material, which is available to authorized users.

## Introduction

Semiconductor colloidal nanocrystals (CNCs) have attracted wide spread attention owing to their intriguing optical properties, which include size- and composition-dependent tunable emission in the whole visible spectrum [1–8]. However, the inherent toxicity of heavy metals (e.g., Cd, Pb, and Te) in CNCs (e.g., CdSe, [8] ZnCdS [9, 10], and ZnCdSe [11]) limits their practical applicability as they contain hazardous and expensive raw materials. Furthermore, the cadmium (Cd-) ions circulate into the biological environment with the passage of time, which restricts their extensive consumption in the biological field [12, 13] and shelters suspicion on their use in CNC or quantum dot-based light-emitting diodes (QD-LEDs). Therefore, it is essentially needed to explore environmental friendly Cd-free nano-emitters for their use in practical applications.

In the last two decades, transition metal ion (e.g., Cu^2+^, Mn^2+^)-doped CNCs have been developed, and they show tunable and efficient photoluminescence (PL) emission [14–17]. The new emission pathways generated by dopant ions result in some additional properties such as large Stokes-shift which can prevent self-absorption or energy transfer [18]. Furthermore, as compared to the undoped and doped binary CNCs (e.g., CdSe, ZnSe:Cu, and CdS:Cu), the subsequent ternary (I/II-III-VI) CNCs demonstrate wider band gap and Stokes-shifted and tunable emission spectrum [11], which majorly depends on stoichiometric ratio of different chemical counter parts [17]. Among various Cd-free doped ternary/quaternary alloyed CNCs, such as Cu:ZnInS [19, 20], Cu:ZnInSe [21], Ag:ZnInSe [22], Mn:ZnInS [16], Mn:CuInS [17], Mn:CuZnInS [23], and Mn:AgZnInS [24], ZnInS has been considered as an ideal candidate to serve as a host because of its wide direct band gap in the visible region and other splendid optical properties [19, 20]. In these ternary CNCs, the dopant ions can replace the host metal ions or stay at an interstitial site. Furthermore, the vacancies and interstitial sites in the crystal structure provide a pathway to the dopant atom [25]. The atomic radii of dopant ion also influence their diffusion into the host CNCs which leads to interstitial/substitutionally doped CNCs [16].

Although the ternary CNCs possess highly efficient and Stokes-shifted emission, the origin of their emission mechanism is very different from the binary doped CNCs [9]. The dominant emission pathways in these ternary CNCs are trap-assisted emission rather than excitonic emission [26]. The introduction of variable amounts of Cu dopant ions in these ternary CNCs transfers them to quaternary CNCs, which leads to a highly Stokes-shifted and dominant dopant-induced emission. Moreover, to enhance the quantum yield (QY) and photo-stability, a nontoxic higher band gap material (ZnS) having small lattice mismatch with Cu-doped Zn-In-S is used as a shell to eliminate the surface trap states and suppress the nonradiative recombination processes. In the last few years because of their visible light tunable and efficient Stokes-shifted emission, these nontoxic CNCs are explored extensively for color conversion applications [20, 21, 27, 28]. However, very recently some work is focused on understanding the origin of this efficient emission and role of different emission pathways and their contribution by variable doping amounts [19, 26]. In the literature, the origin of this Stokes-shifted emission has argued to be coming from the recombination of interstitial and vacancy-assisted donor states [26]. Whereas, similar Cu-doped binary and ternary CNCs (e.g., Cu:CdSe and Cu:ZnCdS) are shown to have a different emission mechanism. For these Cu-doped CNCs, the dopant emission results from the recombination of lower (CB) edge and the dopant state. Furthermore, the change in the composition of these Cu-doped ZnCdS or the size of binary Cu-doped CdSe CNCs shifts conduction bands to lower/higher energy, thus tunes the emission spectrum from visible to NIR region.

In this work, we have synthesized highly efficient Cu-doped ZnInS/ZnS CNCs. The resultant core CNCs possess a broad emission consisting of the variable contributions from deep trap, dopant, and surface state-related emissions. The core CNCs have been passivated by a ZnS shell to remove the surface trap state emission. Additionally, the variation of Zn/In ratios in the core synthesis tunes the emission spectrum from 550 to 650 nm of visible spectrum and has a considerable effect on percentage contribution of different emission pathways. It has been realized that the successful incorporation of Zn ions into the core of quatenery CNCs during the shell growth procedure completely eliminates the zinc vacancy-related emission and, therefore, leads to highly efficient and dominant dopant-induced Stokes-shifted emission. Based on detailed optical studies, the recombination mechanism for these Cu-doped ternary CNCs has been proposed and explained. We have achieved an up to tenfold increase (i.e., from 6.0 to 65.0%) in PL QY after ZnS shell growth on the Cu-doped ZnInS core CNCs. Furthermore, we have studied the generation of white-light emission (WLE) by using different combinations of three distinct Cu-doped CNCs (i.e., possessing green, yellow, and orange emission) with commercially available blue LED as an excitation. The best achieved WLE performance parameters are color coordinate temperature (CCT) 3694 K, luminous efficacy of optical radiation (LER) 170 lm/W_opt_, color rendering index (CRI) 88, and CIE value (0.3330, 0.3125).

## Methods

### Chemicals Used

Zinc acetate (Zn(OAc)_2_; 99.99%), indium acetate (In(OAc)_3_; 99.99%), copper acetate (Cu(OAc)_2_; 99.99%), sulfur powder (S; 99.99%), dodecanethiol (DDT; 98%), oleic acid (OA; 99%), oleylamine (OAm; 70%), and 1-octadecene (ODE; 90%) were purchased from Sigma Aldrich. All the chemicals were used without any further purification.

### Preparation of Stock Solutions

The stock solutions of precursors were prepared before the start of synthesis. For the synthesis of core NCs, Zn, In, Cu, and S stock solutions were prepared. The Zinc (Zn) stock solution (Zn-oleate) was prepared in a three-neck flask. The 0.1 M stock solution of Zn was obtained by dissolving 0.440 g (2 mmol) of Zn(OAc)_2_ in 18.4 mL of ODE and 1.6 mL of OAm and degassing it under vacuum at 95 °C for 30 min. Then, under Argon (Ar) atmosphere, the temperature was raised to 160 °C and kept there for 5 min until a clear solution is obtained. For preparing 0.1 M of In stock solution, 0.584 g (2 mmol) of In(OAc)_2_ was dissolved in 14 mL of ODE and 6 mL of OA. The solution was degassed under vacuum at 95 °C for 30 min. Then the temperature was raised to 160 °C under Ar atmosphere. The solution was retained there for 5 min to obtain a clear solution. The 0.01 M Cu stock solution was prepared by dissolving 0.010 g (0.05 mmol) of Cu (OAc)_2_ in 5.0 mL of OAm at 80 °C in a glove box. The 0.4 M sulfur stock solution (ODE-S) was obtained by dissolving 0.128 g of sulfur powder in 10 mL of ODE by stirring at 140 °C. The Zn stock solution for ZnS shell was prepared by dissolving 1.756 g (8 mmol) of Zn (OAc)_2_ in 6 mL of OAm and 14 mL of ODE. The above solution was degassed under vacuum at 95 °C for 30 min. Then under Argon (Ar) atmosphere, the temperature was raised to 160 °C and kept there for 5 min until a clear solution was obtained. Then these precursors were further used for the synthesis.

### Synthesis of Cu-doped ZnInS Core CNCs

The synthesis was carried out in Ar atmosphere. In the typical procedure, 2 mL of ODE and 1 mL of DDT were added into the three-neck flask. They were kept under vacuum to remove oxygen and water. Then the reaction mixture was purged with Ar. Then 1 mL of 0.1 M Zn-oleate (0.1 mmol), 1 mL of 0.1 M In-oleate (0.1 mmol), 0.5 mL of 0.01 M Cu stock solution (0.01 mmol), and 0.5 mL of 0.4 M ODE-S (0.2 mmol) solution were added to the flask. Then the reaction mixture was heated to 220 °C. The reaction mixture was kept at this temperature for 20 min under Ar flow. The reaction was quenched by immersing the flask in a water bath and cooling it to 60 °C. Ten milliliters of toluene was then added to the mixture. The precipitation of as-synthesized CNCs was done by adding excess ethanol into the toluene solution and centrifuging at 10000 rpm for 10 min. The purification was done by repeated precipitation and re-dispersion of CNCs. The purified CNCs were re-dispersed in toluene for further characterization.

### Deposition of the ZnS Shell over the Core CNCs

The ZnS shell was deposited over the crude Cu-doped ZnInS CNCs. The shell started after 20-min growth time of crude core CNCs. Then the reaction mixture was cooled down to 100 °C, and the shelling process was started. For ZnS shell, 1 mL of 0.4 M stock solution of the Zn precursor was injected into the reaction mixture. After the addition was complete, the reaction temperature was further increased up to 240 °C and kept there for 20 min to allow shell growth. The reaction mixture was then cooled down to 60 °C, and 10 mL of toluene was added at this temperature. The purification method for ZnInS:Cu/ZnS is similar to that of crude CNCs.

### White-Light Emission

To generate white-light emission using the doped core/shell CNCs having different dopant-related PL peak positions, the solid films of the mixed-solution of the CNCs in different compositions were deposited on commercially available quartz-glass wafer using drop-casting method. Then these solid films were integrated over the blue LED emitting at 455 nm, and their optical characterization has been carried out by using integrating sphere and Ocean Optics Maya 2000 spectrometer. The white-light color properties have been calculated by using an in-house written MATLAB code [29].

### Characterization

The absorption spectrum was recorded by using UV-visible spectrophotometer (Varian-Cary 100). The PL emission and PL excitation (PLE) spectra of CNCs were recorded with Cary Eclipse Fluorescence Spectrophotometer. The shape and size of synthesized CNCs were obtained by using a FEI Tecnai Osiris transmission electron microscopy (TEM) operated at 200 kV. X-ray diffraction (XRD) patterns of the CNCs were collected by a XRD spectrometer with a Cu Kα line of 0.15418 nm. Time-correlated single-photon counting (TCSPC) system (Pico-Quant FluoTime 200, Pico-Harp 300) was used for time-resolved fluorescence (TRF) spectroscopy measurements. A picosecond-pulsed laser (Pico-Quant) was used, and the pump intensity was kept low (~ 1 nJ/cm^2^). The measurements were conducted in solution form of CNC samples using quartz cuvettes at room temperature. In order to analyze the PL decay curves, they were fitted with multi-exponential decay functions using Fluo-Fit software in deconvolution mode. The quantum yield (QY) of synthesized CNCs was measured by using de Mello method [30]. A monochromator incorporated with xenon lamp having an excitation wavelength of 400 nm, a Hamamatsu integrating sphere, and an Ocean Optics Maya 2000 spectrometer have been used.

## Results and Discussion

**Fig. 1:**
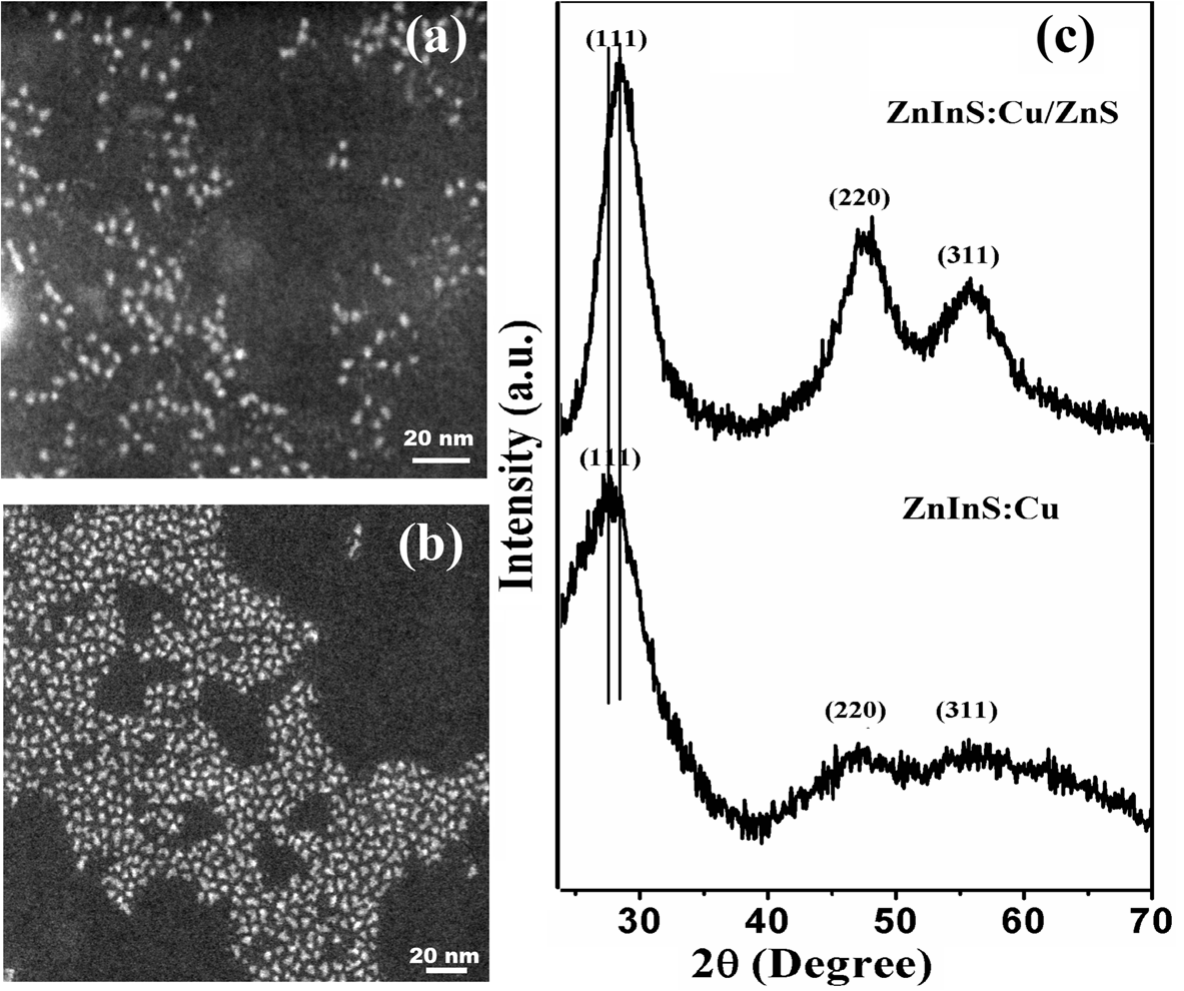
Transmission electron microscopy (TEM) images of **a** ZnInS:Cu (core) and **b** ZnInS:Cu/ZnS (core/shell) CNCs. **c** X-ray diffraction (XRD) pattern of ZnInS:Cu (core) and ZnInS:Cu/ZnS (core/shell) CNCs

Morphological and structural analyses of the as-synthesized CNCs have been performed by using transmission electron spectroscopy (TEM) and XRD studies. TEM images of the synthesized core CNCs (Cu-doped ZnInS) and core/shell (Cu-doped ZnInS/ZnS) CNCs have been demonstrated, respectively, in Fig. 1a, b. From the TEM image of ZnInS:Cu (core, Fig. 1a), it has been analyzed that the particles are nearly spherical in shape and highly monodisperse. Although the CNCs remained monodisperse after the deposition of ZnS shell, the shape of CNCs changed from spherical to triangular. The average size of the synthesized core and core-shell CNCs has been estimated to be 2.50 and 4.48 nm, respectively.

The wide XRD pattern of Cu-doped ZnInS (core) and ZnInS/ZnS (core/shell) CNCs is shown in Fig. 1c. The characteristic peaks demonstrate the zinc blende crystal structure as these peaks have been located between those for cubic ZnS (JCPDS 77–2100) and In_2_S_3_ (JCPDS 05–0731) materials [28, 31]. The XRD pattern does not show any diffraction peaks that arise from Cu. This suggests that the doping does not bring any phase transformation in the crystal structure of the host alloyed NCs. The diffraction peaks appeared at 28.45°, 47.42°, and 55.64° with corresponding (hkl) planes of (111), (220), and (311), respectively. It has been analyzed that the XRD pattern of Cu-doped ZnInS/ZnS core/shell CNCs slightly shifts towards higher angles as compared to Cu-doped ZnInS core CNCs, which may be due to the incorporation of Zn ions in the CNCs [20]. Zn ions have smaller ionic radius as compared to Cu and In ions. Therefore, the diffraction peaks for Cu-doped ZnInS CNCs shift to larger angles after passivating with ZnS shell. The cubic lattice pattern is, however, maintained after the depositing ZnS shell.

**Fig. 2:**
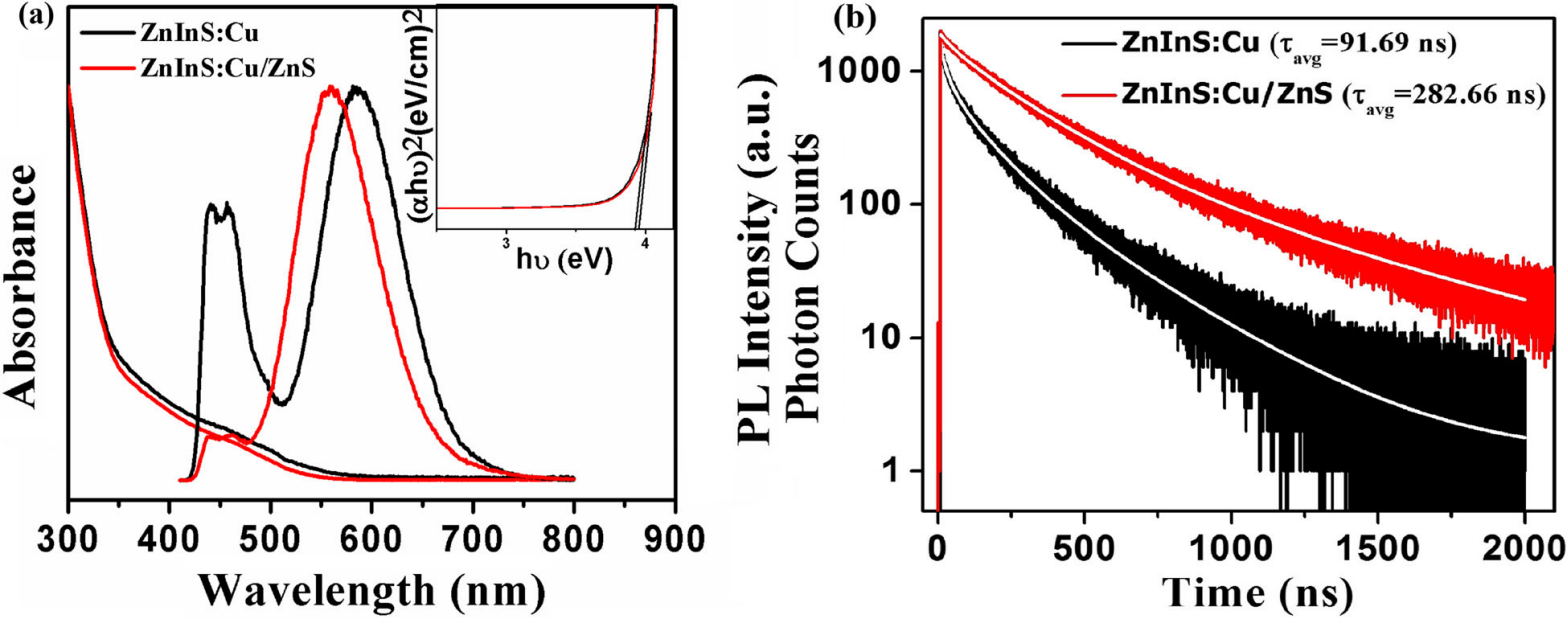
**a** UV-visible absorption and PL emission spectra and **b** PL decay curves of the ZnInS:Cu (core) and ZnInS:Cu/ZnS (core/shell) CNCs. The inset in **a** shows variation of (αE)^1/2^ as a function of the photon energy with shell growth

Absorption and PL spectra of the synthesized core-only and core-shell CNCs have been given in Fig. 2a. These core-only CNCs exhibit an intense defect state PL emission along with a broad Stokes-shifted emission having an overall PL quantum yield (QY) of 6.0%. The broad peak appearing around ~ 450 nm can be ascribed to Zinc interstitial defect state (Zn_i_) and Zinc vacancy (*V*_Zn_) formulated in CNCs [19]. The highly Stokes-shifted emission at ~ 600 nm resembles with typical Cu dopant-induced emission [20]. Similar Stokes-shifted emission has been shown earlier for various Cu-doped binary and ternary CNCs [18, 32, 33]. Furthermore, a large band gap material, ZnS, has been deposited over these core CNCs (Fig. 2a). As evident from PL emission spectra of core-shell CNCs, the broad emission in the range of 450 nm has been suppressed along with proportionate increase in the dopant-related emission. For the best cases, the deposition of ZnS shell on core CNCs results in an increase of the PL QY from 6.0 to 65.0%. After passivating with ZnS shell, the contribution of Cu states dominates the surface defects and traps states [19]. The ZnS has a smaller lattice mismatch with the ZnInS CNCs. Therefore, passivation with ZnS shell allows the gradual strain release, which suppresses the defect state emission and eliminates the surface trap states. In CNCs, the trap states are responsible for nonradiative recombination processes. Thus, deposition of higher band gap ZnS on doped core CNCs lowers the surface defects’ contribution and thereby increases the efficiency of these doped CNCs [19]. Furthermore, after the deposition of shell, the dopant-related emission has been observed to blue-shift with respect to core-only CNCs (Fig. 2a). In the literature, during the shell growth stage, zinc ion diffusion from shell to core region has shown to increase the effective band gap of ternary CNCs which in turn can blue-shift the dopant emission [34]. However, in our case, apart from the blue-shift of dopant emission, there is considerable decrease in broad emission around 450 nm with respect to total integrated emission. Thus, successful Zn ion diffusion into the CNCs may have filled most of the vacancies created by *V*_Zn_. The absorbance spectrum of these core CNCs showed a broad shoulder which is similar to that of typical I-III-VI semiconductor CNCs as observed in previous reports [27, 35, 36]. The absorption spectrum after the deposition of ZnS shell shows a slight blue-shift, which may also be due to the incorporation of more Zn ions into the crystal lattice [34]. This incorporation also leads to a small broadening in the band gap of the core/shell as compared to core-only CNCs (see inset of Fig. 2).

The decay lifetime for these synthesized CNCs was recorded by using FluoTime 200 time-correlated single-photon counting (TCSPC) instrument. The PL decay curves have been fitted by using a multi-exponential decay (Fig. 2b). The amplitude-averaged lifetime of the PL emission at 600 nm for ZnInS:Cu (core) and ZnInS:Cu/ZnS (core/shell) nanocrystals has been calculated as 91.69 and 282.66 ns, respectively. The Cu dopant in core/shell CNCs gives approximately three times larger average lifetime as compared to similar doped core CNCs. This suggests successful elimination of surface defect states by the deposition of ZnS shell over the core CNCs. This result is also supported by ~ 10-fold increase in the absolute QY of core/shell CNCs. The detailed lifetime analysis has been given in supporting information (Additional file 1: Table S1).

**Fig. 3:**
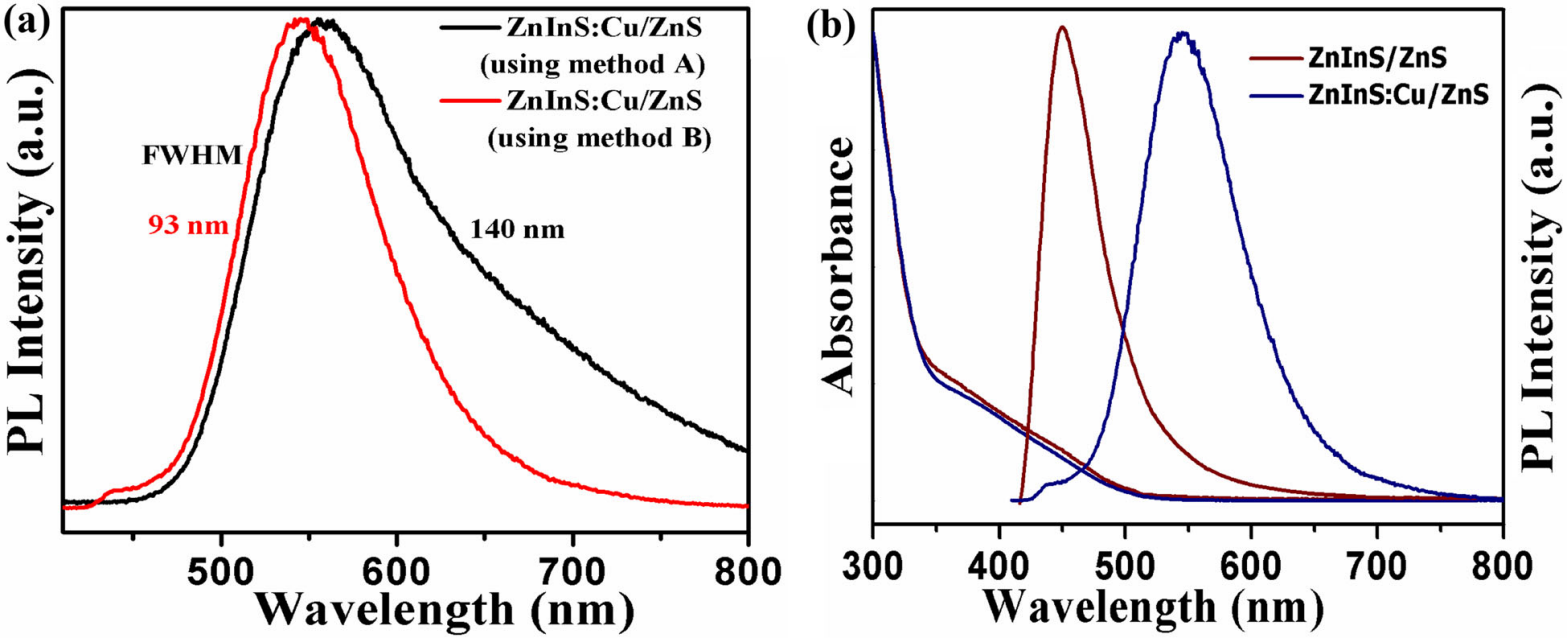
**a** PL emission spectra of ZnInS:Cu/ZnS CNCs synthesized with method A (using powdered indium precursor as previously reported in literature) and method B (using modified method, which used indium oleate as a precursor in this work). **b** PL emission spectra of ZnInS/ZnS (undoped) and ZnInS:Cu/ZnS (doped) CNCs

During the synthesis of colloidal quantum dots (CQDs), the quality of indium precursor is observed to play an important role. When Cu-doped ZnInS/ZnS CNCs are synthesized by using a one-pot method reported previously [20], the resultant PL emission spectra contains a trap state-related PL emission having a long tail at lower energy (Fig. 3a), whereas by making modification in the synthesis recipe and using indium oleate precursor along with other oleate precursors (as explained in experimental section) gives a symmetrical PL emission peak with nearly complete elimination of trap emission in lower energy. Therefore, all presented CNCs discussed here are prepared by using this modified indium precursor. Figure 3b shows the absorbance and PL emission spectrum of doped and undoped CNCs. The absorbance spectrum of Cu-doped ZnInS CNCs shows a slight blue-shift as compared to undoped CNCs. This may be possibly due to a small change in the particle size of these core/shell nanocrystals [37]. For undoped CNCs, the PL emission consists of a broad emission peak around ~ 470 nm. In the literature, the origin of a similar broad emission for these undoped ternary CNCs is believed to be associated with zinc interstitials, vacancies, and their associated deep traps within the band gap [26]. In Fig. 3b, the emission spectrum for the best case of Cu-doped CNCs is also compared, where we observe almost complete suppression of this deep trap-assisted emission along with emergence of a dominant and Stokes-shifted dopant-induced efficient emission.

The UV-visible absorption and PL emission spectra of ZnInS:Cu/ZnS as a function of different Cu concentrations have been shown in Additional file 1: Fig. S1a and S2. Fixed Zn/In concentration was used for studying the effect of variable Cu dopant concentrations. It has been observed that the Cu concentration has a significant effect on the PL emission intensity and peak position. The maximum PL QY of 50.0% was obtained at 2% Cu doping which slightly decreases to 48.0% as the Cu doping increases to 4%. It has been observed that further increase in Cu doping percentage results in the increase of defect states, which further decreases the QY of CNCs (Additional file 1: Fig. S2). However, a small shift in PL peak position occurs by varying Cu concentration which can be attributed to the slight change in the size of CNCs of different Cu concentrations [38].

Photoluminescence excitation (PLE) spectroscopy was used to understand the origin of emission in ZnInS:Cu/ZnS CNCs. The PLE spectrum was collected by exciting the doped CNCs in the wavelength region from 300 to 600 nm at different emission wavelengths of broad dopant emission (i.e., at the peak, red- and blue-tails) as shown in Additional file 1: Fig. S1b. PLE spectra do not show any spectral difference at corresponding emission wavelength. This indicates that the PL emission peak is merely due to Cu dopant, which takes place via energy transfer from the ZnInS host CNCs to Cu dopant states. Furthermore, the overlaid PLE, absorption and PL emission spectra for core/shell CNCs has been shown in Additional file 1: Fig. S3.

**Fig. 4:**
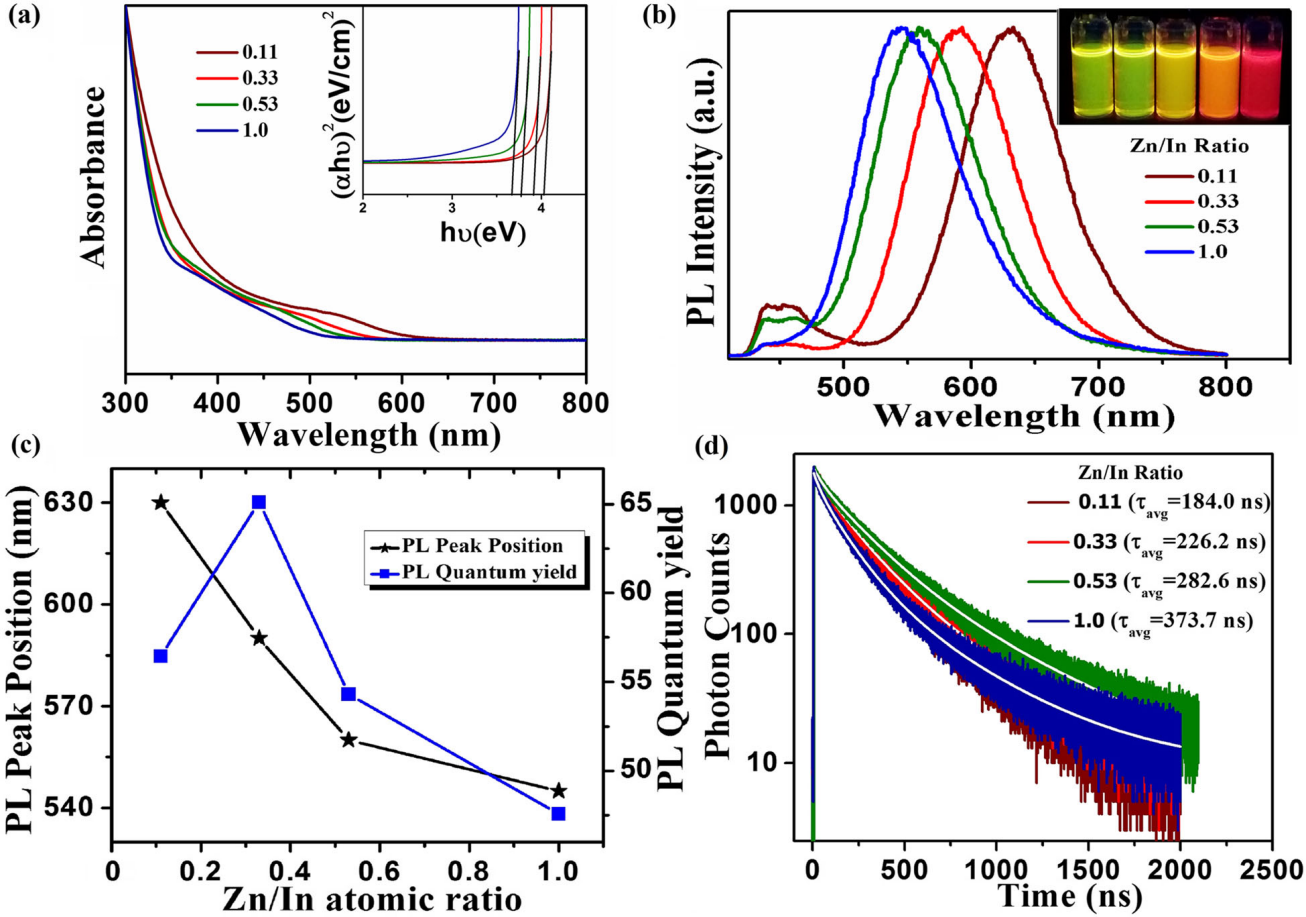
**a** UV-visible absorption and **b** photoluminescence spectra of ZnInS:Cu/ZnS core/shell CNCs as a function of the Zn/In stoichiometric composition. The QY attained for different samples with Zn/In ratio 0.11, 0.33, 0.53, and 1.0 is 56.0, 65.0, 55.0, and 48.0%, respectively. The inset in **a** shows the calculated energy band gap of ZnInS:Cu/ZnS CNCs. **c** Shift of the PL peak position and PL quantum yield with respect to change in Zn/In. **d** PL decay curves of ZnInS:Cu/ZnS CNCs for different Zn/In ratios

Additionally, the PL spectrum has been tuned over the visible region (green to red region) by varying the Zn to In concentration in the reaction mixture. The normalized UV-visible and PL spectra of CNCs have displayed in Fig. 4a, b, respectively. It has been examined that by changing the Zn/In ratio, the energy states of the host semiconductor CNCs are modified, which alter the band gap energy of CNCs. The attained doped ZnInS/ZnS CNCs show tunable band gap which ranges from 3.67 to 4.02 eV (inset of Fig. 4a). Therefore, a continuous tuning in the PL emission spectrum of core/shell CNCs from 550 to 650 nm has been achieved. The broad shoulder in the absorption spectra has been consigned to the electronic transition in the ZnInS host CNCs which experience a considerable blue-shift by increasing the Zn/In stoichiometric ratio. This clearly demonstrates the inclusion of higher band gap ZnS (4.5 eV) in lower band gap InS (2.44 eV), which is also reflected in the absorption spectrum of the alloyed ZnInS CNCs. Figure 4b depicts the corresponding PL spectrum which shows the dependence of the PL peak position on the Zn/In stoichiometric ratio in the resultant Cu-doped ZnInS/ZnS (core/shell) CNCs. This highly Stokes-shifted PL emission from core/shell CNCs with full width at half maxima (FWHM) of ~ 90–110 nm is attributed to dopant-related emission. The Cu d-levels split into Cu T_2_ states and stay above the valence band in the crystal lattice [39]. The electrons localized at the bottom of the conduction band of the host material radiatively recombine with the holes localized in the Cu T_2_ states positioned above the valence band and originate this broad Cu dopant emission [20, 27, 32]. However, in the literature, the origin of this emission for I-III-VI CNCs has been proposed by a recombination of vacancy/interstitial-assisted donor states below the conduction band edge and Cu dopant states which lies above the valence band [39]. However, the tunable PL emission spectrum has been achieved with the alteration in the band gap of host CNCs. The red-shift in the PL peak position is due to the decrease in Zn/In stoichiometric ratio which may change the position of CB edge and can alter the energy difference between CB edge and Cu state. (Fig. 4c).

To further understand the tunable emission behavior of doped CNCs, lifetime decays have been recorded for these core-shell CNCs having different Zn/In ratios (Fig. 4d). The average PL lifetimes have been calculated as 373.7, 282.6, 226.2, and 184.0 ns at the PL emission peak wavelengths of 540, 560, 590, and 630 nm, respectively, for samples possessing different Zn/In ratios (Additional file 1: Tables S2 and S3). The different charge carrier recombination pathways may result in different PL decay lifetimes [40]. However, in literature, the excitonic PL band edge and surface trap emissions provide PL lifetime in the range of few to tens of nanoseconds [41] whereas the lifetime in our case is estimated to be as long as hundreds of nanoseconds for doped CNCs. The increase in Zn/In ratio further increases this lifetime. The long PL lifetimes for doped CNCs are an indication that the PL emission originates from Cu dopant transition rather than surface states of host CNCs. Similar lifetime has been reported for different binary and ternary Cu-doped CNCs [26, 32]. However, increase in the average PL lifetime with increasing Zn/In ratio shows complex nature of this decay pathway which is affected by change in density of different deep trap states and their possible contribution. In these samples, Zn/In ratio has been increased from 0.11 to 1.00 at fixed Cu initial concentrations. In the literature, by considering the valence stability as well as ionic size matching, Cu ions are proposed to occupy Zn sites in ternary CNC lattice [19]. Furthermore, increase of Zn/In ratio can increase interstitial Zinc (Zn_i_) ions in lattice.

**Fig. 5:**
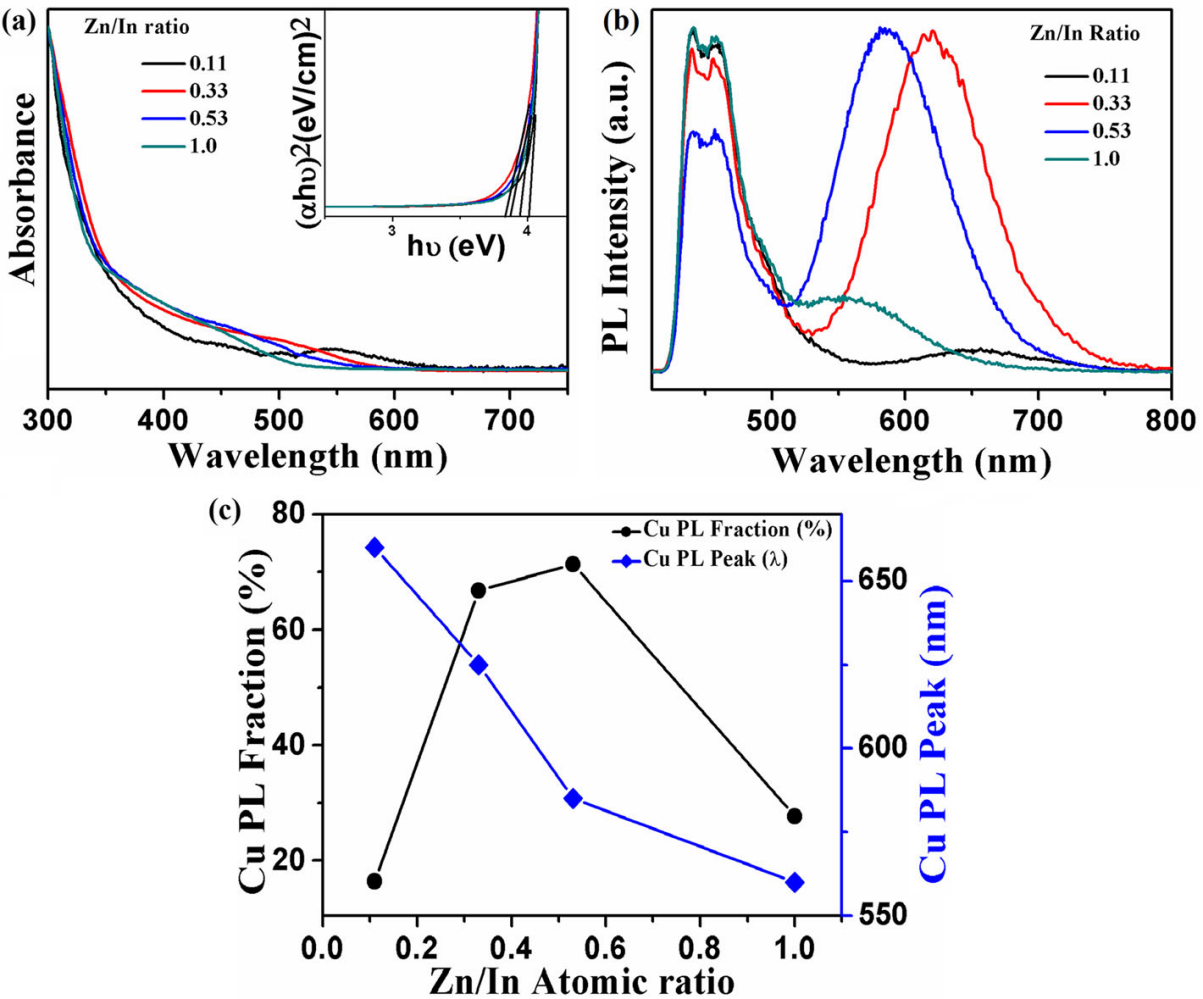
**a** UV-visible absorption and **b** photoluminescence spectra of ZnInS:Cu core CNCs as a function of Zn/In stoichiometric composition. The inset in **a** shows the calculated energy band gap of ZnInS:Cu CNCs. **c** Shift of the Cu dopant PL peak position and Cu contribution (%) with respect to total integrated emission for different CNCs having variable Zn/In ratios

To understand the complex emission mechanism for these Cu-doped ternary CNCs having different stoichiometric ratios, UV-visible and photoluminescence spectra of ZnInS:Cu (core) CNCs with variation in Zn/In ratio have been shown in Fig. 5a, b. Apart from tuning the dopant emission peak position and corresponding band gap, the percentage contribution among deep trap-assisted emission and dopant-induced emission has changed (Fig. 5c). In the literature, a similar increase in Zn/In ratio is proposed to increases the incorporation of Cu ions in the CNCs which improves the emission intensity as a result of increase in radiative recombination from Zn_i_ and In_Zn_ levels to Cu-d states. However, in this study, the decrease of Zn/In ratio is observed to shift the dopant (Cu)-related emission from 550 to 650 nm along with the change in the percentage emission contribution of deep trap-related emission (~ 450 nm) vs dopant emission (550–650 nm). Apart from the large shift in the peak wavelength of dopant emission (~ 100 nm), there is no visible shift in the peak position of deep trap-related emission peaks (~ 450 nm) by changing the Zn/In ratios during the synthesis (Fig. 5b). Therefore, for different Zn/In values, the energies of interstitial zinc and zinc vacancies responsible for this deep trap-related emission (~ 450 nm) remain unchanged within the band gap of host CNCs. Therefore, unlike previous literature reports, where the Zn_i_ (shallow) and In_Zn_ levels act as donor defect levels and Cu ions which substitute the Zn ions and stay above the valence band in CNCs and act as an acceptor level cannot explain our emission mechanism [26]. In the case of binary Cu-doped CdSe CNCs [42] and ternary Zn_*x*_Cd_1 − *x*_S [18], the shift of conduction band edge is shown to tune the Cu-related emission. Furthermore, as shown in Fig. 4b, the shell growth with high band gap ZnS is shown to shift the Cu emission and affect the percentage emission contribution of dopant/deep trap emission. However, there is no considerable shift in deep trap emission position even with the deposition of shell. This result also suggest that the incorporation of Zn ions from shell to core region affects the band gap and tunes the conduction band (CB) edge without having any influence on position of deep trap states. Therefore, different Zn/In values for core Cu-doped Zn-In-S CNCs and zinc diffusion from shell to core region in core-shell CNCs alter the position of CB edge and alter the energy difference between the lowest CB and Cu dopant state which results in these tunable emission spectra.

**Fig. 6:**
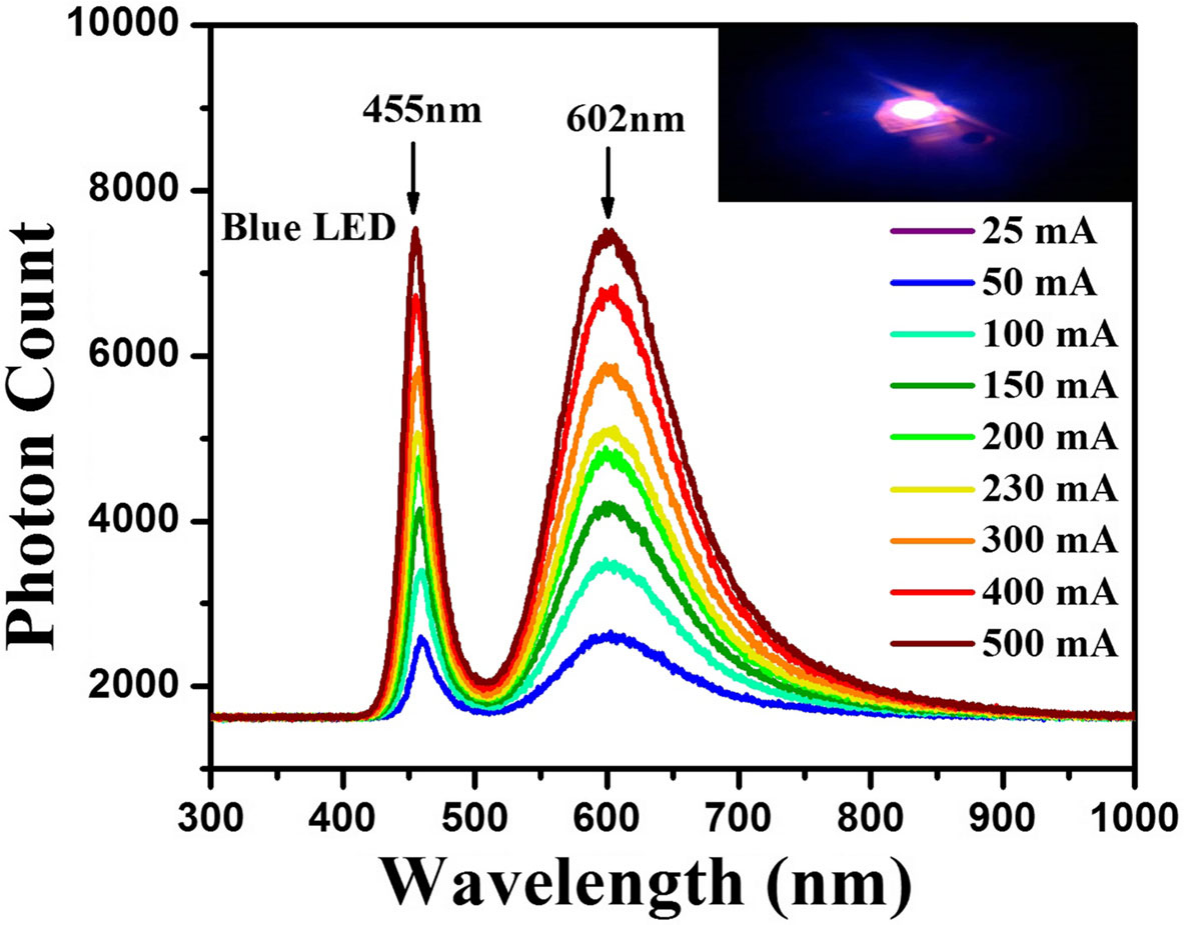
Emission spectra of green (G)-, yellow (Y)-, and orange (O)-emitting ZnInS:Cu/ZnS CNCs integrated on LED driven at varying current injection levels

As a proof-of-concert demonstration, these highly efficient Cu-doped ZnInS/ZnS CNCs having PL emissions from green to red region have been tested to generate white-light emission (WLE) by integrating their broad dopant-related PL emission with commercial blue LEDs. The calculated parameters depict that the obtained WLE exhibit a good performance. Also, it has been noticed that the undoped CNCs possess low CRI (< 80) value as the PL emission spectrum is not wider. However, WLEDs fabricated by using a single-type Cu-doped CNCs also possess low CRI (Additional file 1: Table S4). Furthermore, the WLEDs for indoor applications should satisfy the specific requirements (CRI > 80, LER > 350 lm/W_opt_, CCT < 4500 K) [43]. In order to meet all these requirements, we have used different combinations of CNCs to generate WLE (listed in Table 1, Additional file 1: Tables S4 and S5). In order to get white-light emission, a film of CNCs emitting at different wavelengths has been prepared on the commercially available quartz-glass wafer using drop-casting method and integrated it over the blue LED emitting at 455 nm. The obtained emission spectra for different forward currents ranging from 25 to 500 mA have been presented in Fig. 6, Additional file 1: Figs. S4 and S5. In order to evaluate the quality of emitted light, different device parameters were calculated which are given in Table 1, Additional file 1: Tables S4 and S5. It has been observed that combining a blue LED with green (G)-, yellow (Y)-, and orange (O)-emitting CNCs, with more weight of G-emitting CNCs (i.e., G/Y/O ratio is 15/1/0.75), yields better results than other combinations. The best achieved CIE color coordinates are (0.333, 0.3125) on the CIE 1931 chromaticity diagram. Thus, it covers the white-light region and is close to the equi-energy white point (0.3333, 0.3333). A large amount of G-emitting CNCs is used because of less absorption for these CNCs by 455-nm blue LED. Figure 4a shows that these G-emitting CNCs possess blue-shifted absorption spectrum as compared to Y- and O-emitting CNCs. Therefore, more amounts of G-emitting CNCs were used to increase green component in resultant emission spectrum. It is important to mention here that due to a large Stokes-shift in these Cu-doped CNCs, the increase in the amount of a particular color (green for our case) component will not result in the decrease in the final color output due to negative re-absorption effects.

**Table 1:** CRI, luminous efficacy of optical radiation (LER), CCT, and CIE color coordinates of the white-light emission based on G-, Y-, and O-emitting Cu:ZnInS/ZnS CNCs blends with different weight ratios operated at different current levels

Current	Optical Power	LER	Lumens	CRI	CQS	CCT	*X*	*Y*
25 mA	0.4597	163.59	75.20	88.59	95.56	6093.36	0.3218	0.3126
50 mA	0.4978	170.95	85.10	87.72	92.79	5454.43	0.3330	0.3125
100 mA	0.5661	181.52	102.77	88.11	89.63	4721.94	0.3471	0.3123
150 mA	0.6216	188.12	116.93	87.00	86.47	4350.70	0.3546	0.3108
200 mA	0.6732	193.07	129.96	85.85	84.19	4115.00	0.3596	0.3095
230 mA	0.6984	195.02	136.20	85.42	83.21	4058.48	0.3605	0.3081
300 mA	0.7588	199.01	151.00	84.03	81.09	3871.06	0.3642	0.3058
400 mA	0.8359	203.83	170.37	82.53	78.55	3693.66	0.3679	0.3040
500 mA	0.8988	206.15	185.28	81.40	76.99	3607.27	0.3691	0.3011

The calculated LER was in the range of 170–200 lm/W_opt_ which defines the range of color sensitivity. The color rendition performance has a CQS value of 95, which indicates its good color rendition capability. The CCT value is between 3694 and 5454 K. The highest CRI is 88, suggesting these can be favorably used for indoor applications. The device parameters obtained from other combinations (listed in Additional file 1: Tables S4 and S5) are not optimum. When using G- and O-emitting CNCs with blue LED, it gives CIE (0.3128, 0.2989), CCT 6799–6307 K, CRI 87, and LER 158–165 lm/W_opt_. The next combination was tried with G-, Y-, and O-emitting CNCs with blue LED, which gives CIE (0.3184, 0.3066), CCT 4114–6337 K, CRI 88, and LER 160–175 lm/W_opt_. Therefore, increasing the weight ratio by adding more amounts of G-emitting CNCs with appropriate Y- and O-emitting CNCs provides good results by eliminating the green window problem. It concludes that the Stokes-shifted emission intensity from different colors in a multiphase emitter-based WLED has great impact on quality of light.

## Conclusions

The high quantum yield Cu-doped ZnInS/ZnS CNCs have been synthesized by using a modified synthesis route. The resultant CNCs possess nearly defect-free and symmetric emission. The optical band gap has been tuned (3.67 to 4.02 eV) by variation in Zn/In ratio. The highly efficient and Stokes-shifted emission has been varied from green to red region and possesses a high quantum yield of 65.0%. Time-resolved PL decay curves with decay time of hundreds of nanoseconds indicate that the dominant emission is achieved by the introduction of dopant ions. The origin of different deep traps and their densities are shown to have no considerable effect in tuning the Cu-related emission, and the origin of dopant-related emission has been understood in detail using different optical studies. At last, the synthesized G-, Y-, O-, and R-emitting CNCs with different combinations have been used to generate white-light emission. The best white-light emission results are obtained by combining G-, Y-, and O-emitting CNCs in suitable weight ratios. These performance metrics and detailed photo-physical studies show that these Cd-free Cu-doped ZnInS/ZnS core/shell CNCs can be used in a variety of applications including lighting and displays.

## Additional file

Additional file 1: **Figure S1.** (a) UV-visible and PL spectrum of ZnInS:Cu/ZnS CNCs by various Cu doping amounts. (b) PLE spectrum of ZnInS:Cu/ZnS CNCs at different emission wavelengths (500, 550, and 600 nm) acquired from PL spectrum (inset). **Figure S2.** UV-visible and PL spectrum of ZnInS:Cu (core) and ZnInS:Cu/ZnS (core/shell) CNCs with different Cu dopant percentages. **Figure S3.** UV-visible, PL, and PLE spectrum of ZnInS:Cu/ZnS CNCs. **Table S1.** Fluorescence decay components of the Cu-doped ZnInS (core) and ZnInS/ZnS (core/ shell) CNCs. **Table S2.** Fluorescence decay components of the Cu-doped ZnInS/ZnS CNCs. **Table S3.** Fluorescence decay components of the Cu-doped ZnInS/ZnS CNCs. **Figure S4.** EL spectra of G- and O-emitting ZnInS:Cu/ZnS CNCs integrated LED. **Table S4.** The CRI, luminous efficacy of optical radiation (LER), CCT, and CIE color coordinates of the as-fabricated WLEDs based on G- and O-Cu:ZnInS/ZnS CNCs blends with different weight ratios operated at different currents (mA). Figure S5. EL spectra of G-, Y-, O-emitting ZnInS:Cu/ZnS CNCs integrated LED. Table S5. The CRI, luminous efficacy of optical radiation (LER), CCT, and CIE color coordinates of the as-fabricated WLEDs based on G-, Y-, and O- Cu:ZnInS/ZnS CNC blends with different weight ratios operated at different currents (mA). (DOCX 1212 kb)

## Abbreviations

CB: Conduction band
CCT: Color coordinate temperature
CIE: Commission Internationale de l’Enclairage
CNCs: Colloidal nanocrystals
CQDs: Colloidal quantum dots
CRI: Color rendering index
FWHM: Full width at half maxima
LER: Luminous efficacy of optical radiation
PL: Photoluminescence
PLE: Photoluminescence excitation
QD-LED: Quantum dot-based light-emitting diode
QY: Quantum yield
TCSPC: Time-correlated single photon-counting
TEM: Transmission electron microscopy
TRF: Time-resolved fluorescence
*V*_Zn_: Zinc vacancy
WLE: White-light emission
XRD: X-ray diffraction
Zn_i_: Zinc interstitial
