# Supplementary material for: Cd-free Cu-doped ZnInS/ZnS Core/Shell Nanocrystals: Controlled Synthesis And Photophysical Properties
Source: Nanoscale Res Lett. 2018 Jun 18;13:182. doi: 10.1186/s11671-018-2599-x (PMC6006007; doi:10.1186/s11671-018-2599-x)
Supplement: Supplementary file 1 — Figure S1. (a) UV-visible and PL spectrum of ZnInS:Cu/ZnS CNCs by various Cu doping amounts. (b) PLE spectrum of ZnInS:Cu/ZnS CNCs at different emission wavelengths (500, 550, and 600 nm) acquired from PL spectrum (inset). Figure S2. UV-visible and PL spectrum of ZnInS:Cu (core) and ZnInS:Cu/ZnS (core/shell) CNCs with different Cu dopant percentages. Figure S3. UV-visible, PL, and PLE spectrum of ZnInS:Cu/ZnS CNCs. Table S1. Fluorescence decay components of the Cu-doped ZnInS (core) and ZnInS/ZnS (core/ shell) CNCs. Table S2. Fluorescence decay components of the Cu-doped ZnInS/ZnS CNCs. Table S3. Fluorescence decay components of the Cu-doped ZnInS/ZnS CNCs. Figure S4. EL spectra of G- and O-emitting ZnInS:Cu/ZnS CNCs integrated LED. Table S4. The CRI, luminous efficacy of optical radiation (LER), CCT, and CIE color coordinates of the as-fabricated WLEDs based on G- and O-Cu:ZnInS/ZnS CNCs blends with different weight ratios operated at different currents (mA). Figure S5. EL spectra of G-, Y-, O-emitting ZnInS:Cu/ZnS CNCs integrated LED. Table S5. The CRI, luminous efficacy of optical radiation (LER), CCT, and CIE color coordinates of the as-fabricated WLEDs based on G-, Y-, and O- Cu:ZnInS/ZnS CNC blends with different weight ratios operated at different currents (mA). (DOCX 1212 kb) [file 11671_2018_2599_MOESM1_ESM.docx]

**Supporting Information**

**Cd-free Cu-doped ZnInS/ZnS Core/Shell Nanocrystals: Controlled Synthesis and Photo-physical Properties**

Manpreet Kaur^1,2^, Ashma Sharma^2,3^, Murat Olutas^2,4^, Onur Erdem^2^, Akshay Kumar^1^, Manoj Sharma^1,2,3*^, Hilmi Volkan Demir^2,3*^

^1^Department of Nanotechnology, Sri Guru Granth Sahib World University, Punjab 140406, India.

^2^Department of Electrical and Electronics Engineering, Department of Physics, and UNAM–Institute of Materials Science and Nanotechnology, Bilkent University, Ankara 06800, Turkey.

^3^LUMINOUS! Center of Excellence for Semiconductor Lighting and Displays, School of Electrical and Electronics Engineering, School of Physical and Mathematical Sciences, School of Materials Science and Engineering, Nanyang Technological University, Nanyang Avenue 639798, Singapore.

^4^Abant Izzet Baysal University, Department of Physics, Bolu 14030, Turkey

*Corresponding Authors:

Email: [hvdemir@ntu.edu.sg](mailto:hvdemir@ntu.edu.sg),

m.sharma@ntu.edu.sg

*
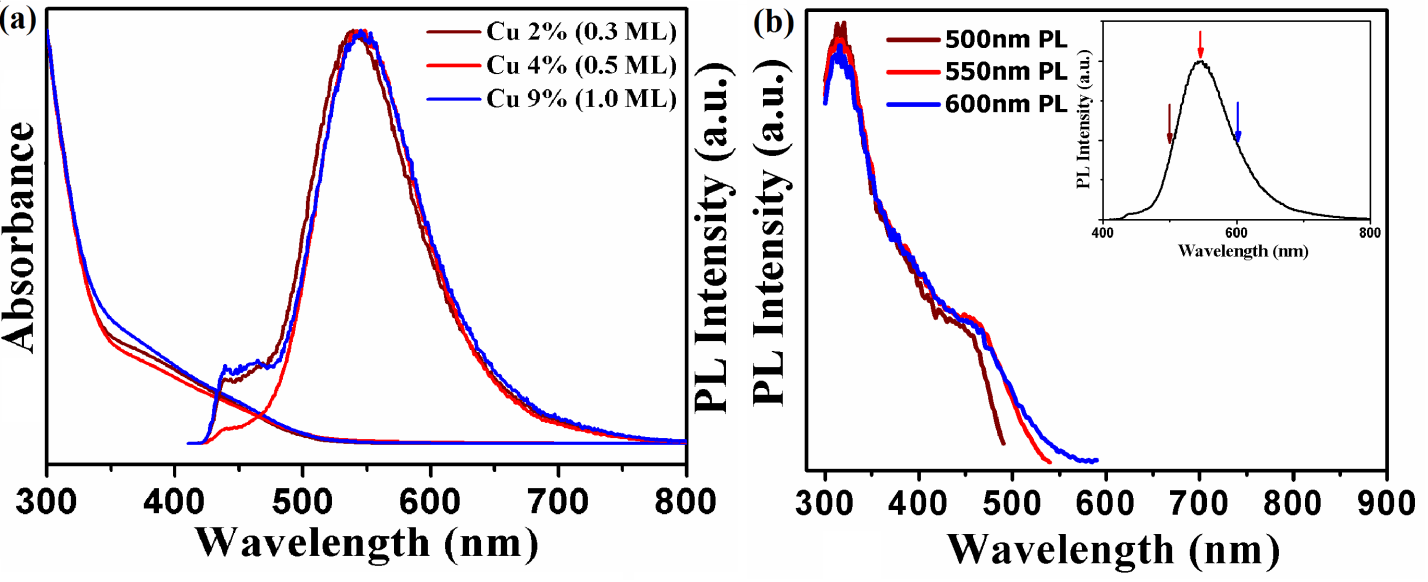
*

**Figure S1.** (a) UV-visible and PL spectrum of ZnInS:Cu/ZnS CNCs by various Cu doping amount. (b) PLE spectrum of ZnInS:Cu/ZnS CNCs at different emission wavelengths (500 nm, 550 nm and 600 nm) acquired from PL spectrum (inset).


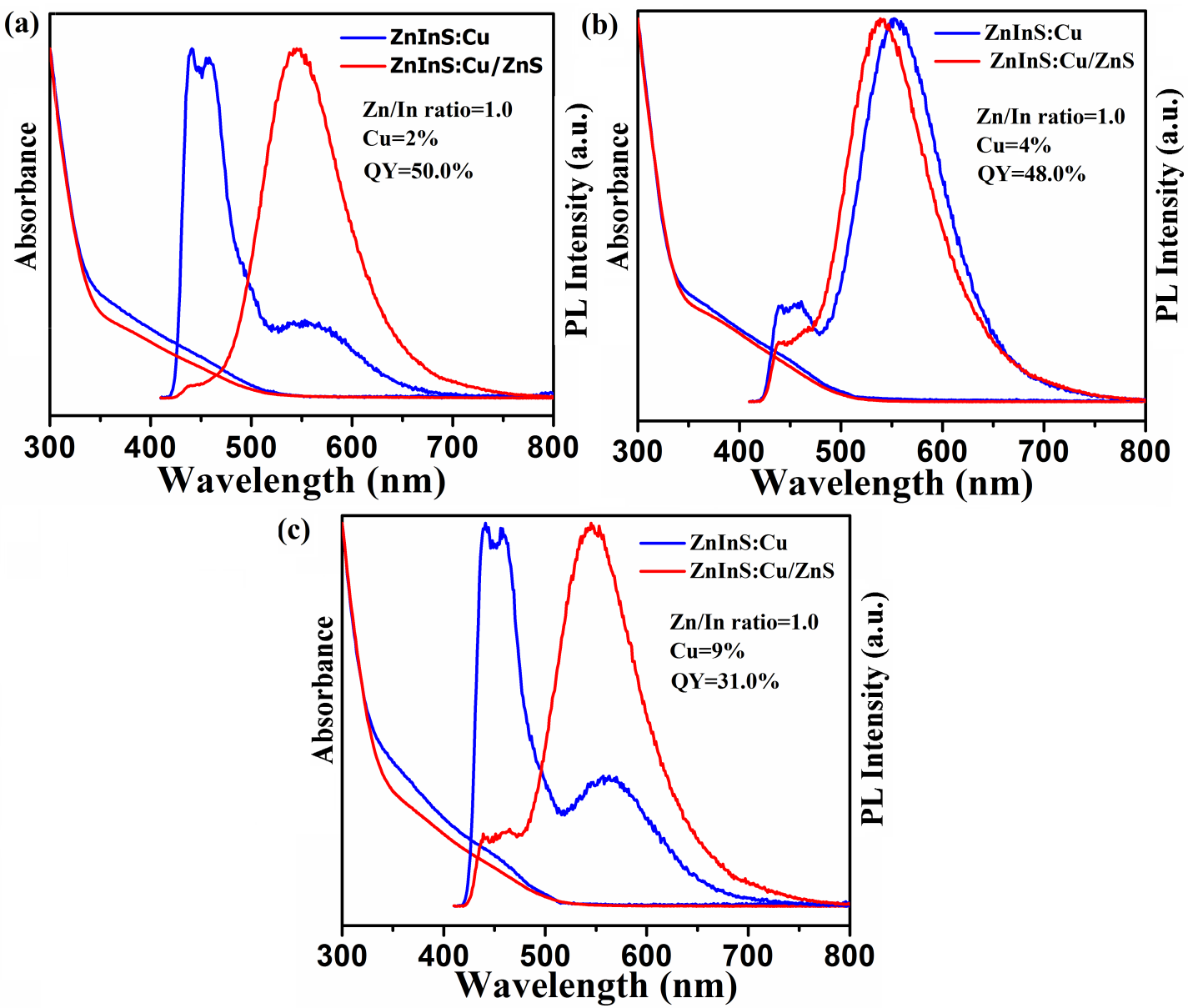


**Figure S2.** UV-visible and PL spectrum of ZnInS:Cu (core) and ZnInS:Cu/ZnS (core/shell) CNCs with different Cu dopant percentage.


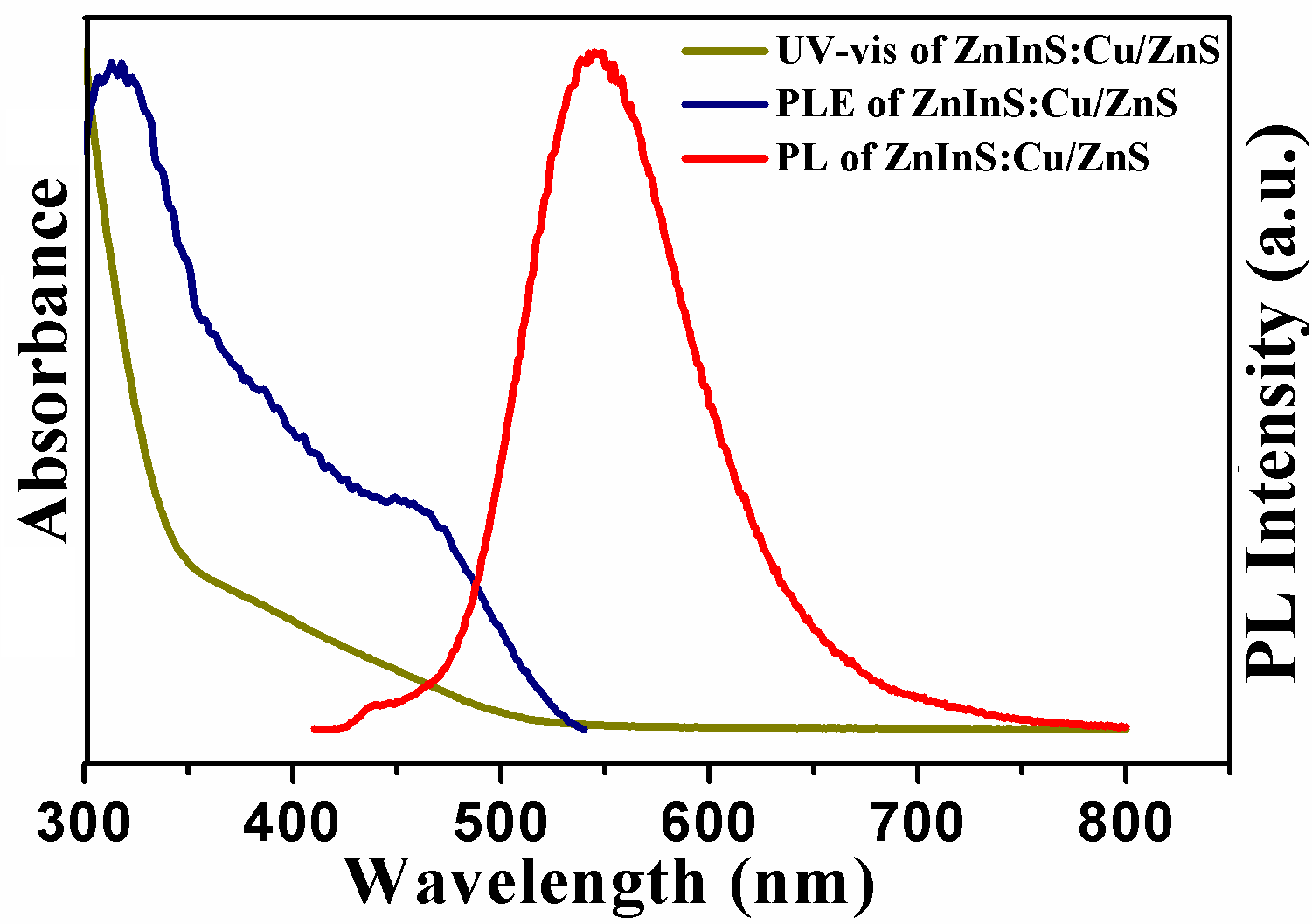


**Figure S3.** UV-visible, PL, PLE spectrum of ZnInS:Cu/ZnS CNCs.

| **Samples** | **TRF Decay Components** | | | | | | | | **Amplitude Average lifetime**  $\frac{\sum\boldsymbol{A}_{\boldsymbol{i}}\boldsymbol{\tau}_{\boldsymbol{i}}}{\sum\boldsymbol{A}_{\boldsymbol{i}}}$ |
| --- | --- | --- | --- | --- | --- | --- | --- | --- | --- |
|  | **τ_1_ (ns)** | **τ_2_ (ns)** | **τ_3_ (ns)** | **Τ_4_ (ns)** | **A_1_** | **A_2_** | **A_3_** | **A_4_** | **τ_avg_ (ns)** |
| ZnInS:Cu | 316 | 123 | 28.4 | 4.4 | 254 | 594 | 554 | 464 | 91.69 |
| ZnInS:Cu/ZnS | 661 | 236 | 44.8 | 0 | 326 | 1287 | 266 | 0 | 282.66 |

**Table S1.** Fluorescence decay components of the Cu-doped ZnInS (core) and ZnInS/ZnS (core/ shell) CNCs.

**Table S2.** Fluorescence decay components of the Cu-doped ZnInS/ZnS CNCs.

| **Zn/In (nominal ratio)** | **TRF Decay Components** | | | | | | **Amplitude Average lifetime**  $\frac{\sum\boldsymbol{A}_{\boldsymbol{i}}\boldsymbol{\tau}_{\boldsymbol{i}}}{\sum\boldsymbol{A}_{\boldsymbol{i}}}$ |
| --- | --- | --- | --- | --- | --- | --- | --- |
|  | **τ_1_ (ns)** | **τ_2_ (ns)** | **τ_3_ (ns)** | **A_1_** | **A_2_** | **A_3_** | **τ_avg_ (ns)** |
| 0.11 | 377 | 152 | 29.9 | 458 | 1039 | 357 | 184.07 |
| 0.33 | 417 | 177 | 36.2 | 532 | 1106 | 247 | 226.28 |
| 0.53 | 661 | 236 | 44.8 | 326 | 1287 | 266 | 282.66 |
| 1.0 | 900 | 298 | 65.8 | 331 | 1158 | 281 | 373.71 |

| **Zn/In**  **(nominal**  **ratio)** | **Fractional Emission Contributions**  $\frac{\boldsymbol{A}_{\boldsymbol{i}}\boldsymbol{\tau}_{\boldsymbol{i}}}{\sum\boldsymbol{A}_{\boldsymbol{i}}\boldsymbol{\tau}_{\boldsymbol{i}}}$ | | | | | |
| --- | --- | --- | --- | --- | --- | --- |
|  | **A_1_τ_1_** | **A_2_τ_2_** | **A_3_τ_3_** | **A_1_τ_1_ (%)** | **A_2_τ_2_ (%)** | **A_3_τ_3_ (%)** |
| 0.11 | 172666 | 157928 | 10674.3 | 50.59538 | 46.27679 | 3.12783 |
| 0.33 | 221844 | 195762 | 8941.4 | 52.00923 | 45.89455 | 2.09623 |
| 0.53 | 215486 | 303732 | 11916.8 | 40.57087 | 57.18548 | 2.24365 |
| 1.0 | 297900 | 345084 | 18489.8 | 45.0358 | 52.16896 | 2.79524 |

**Table S3.** Fluorescence decay components of the Cu-doped ZnInS/ZnS CNCs.


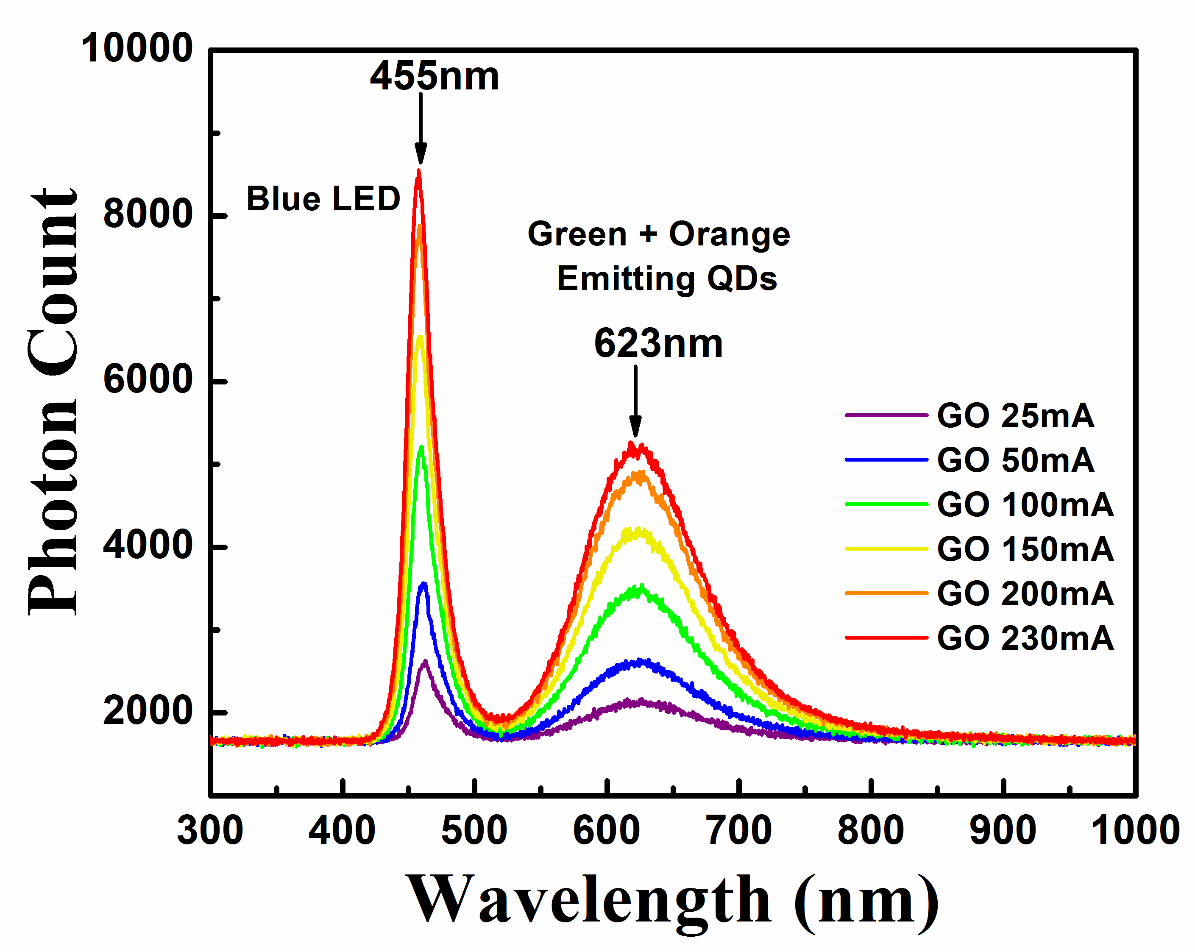


**Figure S4.** EL spectra of G- O- emitting ZnInS:Cu/ZnS CNCs integrated LED.

**Table S4.** The CRI, luminous efficacy of optical radiation (LER), CCT, and CIE color coordinates of the as-fabricated WLEDs based on G- and O- Cu:ZnInS/ZnS CNCs blends with different weight ratios operated at different currents (mA).

| **Blue LED + Green +Orange emitting CNCs** | | | | | | | | |
| --- | --- | --- | --- | --- | --- | --- | --- | --- |
| Current | Optical Power | LER | Lumens | CRI | CQS | CCT | X | Y |
| 25 mA | 0.4749 | 157.79 | 74.93 | 87.86 | 95.63 | 6799.47 | 0.3128 | 0.2989 |
| 50 mA | 0.5192 | 159.53 | 82.82 | 84.68 | 93.06 | 6640.71 | 0.3167 | 0.2878 |
| 100 mA | 0.6002 | 162.22 | 97.35 | 78.43 | 89.01 | 6409.53 | 0.3215 | 0.2731 |
| 150 mA | 0.6685 | 164.24 | 109.80 | 74.12 | 86.06 | 6306.90 | 0.3237 | 0.2637 |
| 200 mA | 0.7335 | 165.11 | 121.11 | 70.96 | 83.57 | 6228.04 | 0.3252 | 0.2567 |
| 230 mA | 0.7657 | 164.98 | 126.32 | 68.72 | 82.20 | 6307.34 | 0.3250 | 0.2521 |


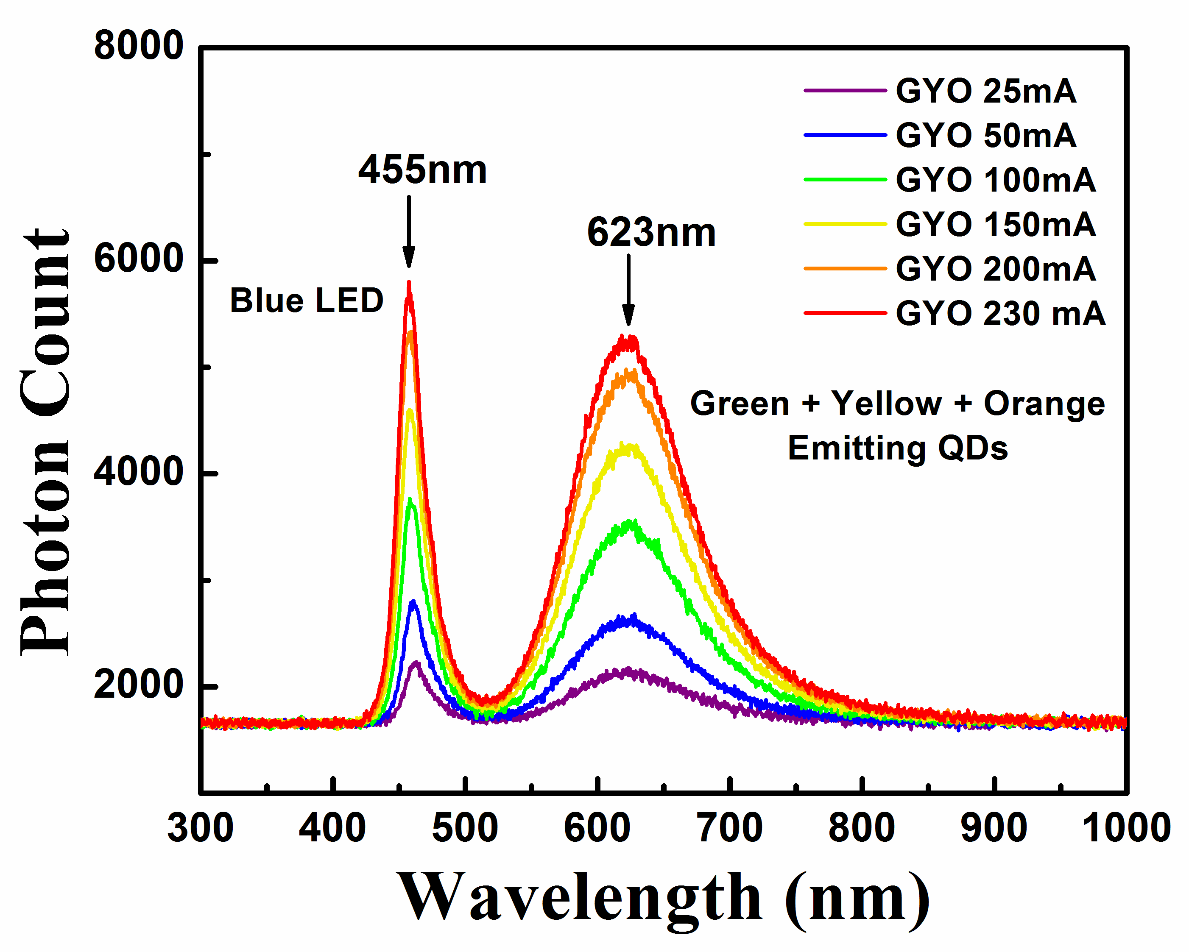


**Figure S5.** EL spectra of G- Y- O- emitting ZnInS:Cu/ZnS CNCs integrated LED.

**Table S5.** The CRI, luminous efficacy of optical radiation (LER), CCT, and CIE color coordinates of the as-fabricated WLEDs based on G- and Y- and O- Cu:ZnInS/ZnS CNC blends with different weight ratios operated at different currents (mA).

| **Blue LED + Green + Yellow + Orange emitting CNCs** | | | | | | | | |
| --- | --- | --- | --- | --- | --- | --- | --- | --- |
| Current | Optical Power | LER | Lumens | CRI | CQS | CCT | X | Y |
| 25 mA | 0.4684 | 159.18 | 74.56 | 88.71 | 96.16 | 6337.92 | 0.3184 | 0.3066 |
| 50 mA | 0.5070 | 162.69 | 82.48 | 86.81 | 94.24 | 5762.27 | 0.3279 | 0.3016 |
| 100 mA | 0.5761 | 167.32 | 96.39 | 83.17 | 91.36 | 5001.07 | 0.3400 | 0.2950 |
| 150 mA | 0.6363 | 171.62 | 109.19 | 79.24 | 90.25 | 4498.06 | 0.3479 | 0.2906 |
| 200 mA | 0.6901 | 173.59 | 119.79 | 77.68 | 88.56 | 4246.55 | 0.3513 | 0.2857 |
| 230 mA | 0.7181 | 174.67 | 125.43 | 77.02 | 87.84 | 4114.44 | 0.3531 | 0.2837 |
